# Analysis of Neutrophil/Lymphocyte Ratio as a Potential Biomarker Stratified by Breast Cancer Histologic Subtype

**DOI:** 10.3390/diagnostics16030449

**Published:** 2026-02-01

**Authors:** Emily Hunt, Matthew Davis, Wei Hou, Henrietta Bains, Timothy Darby, Julia Hou, Julie Chung, Roham Hadidchi, Tim Q. Duong, Takouhie Maldjian

**Affiliations:** Department of Radiology, Montefiore Medical Center, Albert Einstein College of Medicine, Bronx, NY 10467, USA; matthew.davis@einsteinmed.edu (M.D.); weihouanhui@gmail.com (W.H.); henrietta.bains@alumni.einsteinmed.edu (H.B.); julia.hou@einsteinmed.edu (J.H.); juchung@montefiore.org (J.C.); roham.hadidchi@einsteinmed.edu (R.H.); tim.duong@einsteinmed.edu (T.Q.D.)

**Keywords:** neutrophil/lymphocyte ratio, background parenchymal enhancement, breast cancer, invasive ductal carcinoma, invasive lobular carcinoma, biomarkers, radiomics

## Abstract

**Background/Objectives**: Breast cancer is the most common cancer in women. The neutrophil/lymphocyte ratio (NLR) is an emerging biomarker from peripheral blood that has been associated with breast cancer prognosis in some studies; however, some studies fail to demonstrate an association. We stratified breast cancer patients into invasive lobular carcinoma (ILC) and invasive ductal carcinoma (IDC) cohorts to evaluate if any meaningful association could be found in either cohort between NLR and mortality. Additionally, no prior studies have examined the relationship between NLR and background parenchymal enhancement (BPE) on breast MRI, an imaging feature linked to increased breast cancer risk and a potential imaging prognostic biomarker, so we examined the relationship between BPE and NLR in the two cohorts. **Methods**: This retrospective study included 794 breast cancer patients who had either IDC or ILC. Radiologists’ MRI reports and their BI-RADS categorization of BPE (1 = minimal, 2 = mild, 3 = moderate, 4 = marked) were extracted and recorded. The NLR was calculated from blood counts obtained prior to treatment. Tumor characteristics were also recorded. **Results**: For patients with ILC, NLR was found to be associated with mortality. Additionally, patients with ILC and a high BPE had a significantly higher mean NLR compared to all other groups, including low BPE groups and all IDC groups. **Conclusions**: There is potential value in using NLR, a readily available blood biomarker, in models predicting prognosis in ILC patients.

## 1. Introduction

Accurate and noninvasive prognostic tools are valuable for enhancing cancer risk stratification and patient care. Traditionally, tumor stage and hormone receptor status have guided cancer management and prognosis. In recent years, imaging and blood-based biomarkers have gained attention as a potential tool to reflect tumor aggressiveness [1,2]. Given that breast cancer is the most common cancer in women and the second leading cause of cancer-related death among women in the United States [3], these novel advancements offer opportunities for the investigation and development of accessible, non-invasive, and cost-effective biomarkers that can optimize risk stratification and inform clinical decision-making for breast cancer patients.

The neutrophil/lymphocyte ratio (NLR) is a potential biomarker that has been increasingly studied in the literature. The NLR is a measurement of the ratio between the absolute neutrophil and lymphocyte counts in peripheral blood. The NLR can be obtained in routine and widely available blood tests, which makes its adoption as a prognostic tool convenient. Several studies have investigated the role of NLR in breast cancer prognosis. Elevated NLR has been associated with poorer prognoses, including lymph node involvement, metastasis, pathologic complete response (pCR), and overall survival in various clinical contexts [4,5,6,7,8,9,10,11,12,13,14]. However, findings have been inconsistent in the literature. For example, one study reported that while NLR predicted all-cause mortality, it did not correlate with breast-cancer-specific mortality in non-metastatic breast cancer patients [15]. Additionally, a separate study concluded that NLR should not be considered as a reliable prognostic marker in early breast cancer [16].

A factor that may contribute to these inconsistencies in the literature possibly stems from failure to account for breast cancer subtypes, which exhibit biologically variable profiles and patterns of behavior. Invasive ductal carcinoma (IDC) and invasive lobular carcinoma (ILC) are the two most common breast cancer pathologies, but they differ in their histology, metastatic behavior, and receptor status. IDC is the most common subtype, making up approximately 80% of breast cancer diagnoses, while ILC is less common but has a distinct growth and metastatic pattern and is more frequently hormone-receptor positive [17]. Considering these biological and clinical differences, there should be careful separation of them in making meaningful subtype-specific associations in prognosis and outcomes in breast cancer research.

Background parenchymal enhancement (BPE) is an imaging feature seen on contrast-enhanced breast MRI. BPE represents enhancement of normal fibroglandular tissues and is influenced by age, breast density, and hormones. Similar to NLR, higher BPE has been associated with increased risk of breast cancer, and thus BPE may serve as a prognostic tool [18,19]. Although both BPE and NLR have shown prognostic potential, the relationship between them has previously never been studied.

To our knowledge, there have been no studies to date that have directly examined the relationship between NLR and BPE, nor investigated how NLR and its relationship to BPE and survival may differ along breast cancer subtypes, specifically IDC and ILC. In addition, no prior study has investigated the association between clinical parameters of prognostic significance and NLR while stratifying by IDC versus ILC. This study aims to address this knowledge gap, as subtype-specific differences may explain differing results in the literature regarding NLR as a tool for evaluating prognosis.

The present retrospective study aimed to investigate the association between NLR and mortality and separately NLR with BPE, with the goal of investigating the utility of using a readily available blood-based biomarker for predicting prognosis in breast cancer patients with IDC or ILC, which may help guide treatment and promote personalized care. We hypothesized that patterns of association between NLR, BPE, and mortality will differ between IDC and ILC.

## 2. Materials and Methods

In this retrospective study, we identified patients from our institution’s Tumor Registry database diagnosed with invasive breast cancer between 1 January 2016 and 30 April 2023. Included patients were those with either IDC or ILC who underwent a pre-treatment breast MRI. Patients with mixed histology, such as concurrent IDC and ILC, or incomplete records, such as no BPE or NLR values available, were excluded. The final study cohort consisted of 794 patients.

This study was approved by the Institutional Review Board with a waiver of informed consent (IRB REF#105846 date 21 September 2023). All data were derived between 1 January 2016 to 30 April 2023 from the electronic medical records (EMRs) within our health system, which includes multiple hospitals and outpatient clinics serving an urban area. Data extraction was performed using the Observational Medical Outcomes Partnership (OMOP) common data model, as previously described [20,21], which standardizes clinical data extracted from the EMR into a research-ready format. In addition to a dedicated team of data scientists and engineers performing data extraction and validation, manual chart review was routinely performed on subsets of patients to confirm the accuracy of key variables. Based on these validation efforts, we expect misclassification or measurement error in NLR to be minimal.

Breast cancer outcomes, characteristics, and mortality data were obtained from our institution’s cancer registry, which serves as the authoritative source for oncologic diagnoses, staging, and vital status. As a National Cancer Institute-designated Comprehensive Cancer Center, our institution is subject to standardized reporting, auditing, and data-sharing requirements, making relevant misclassification in outcome ascertainment unlikely.

BPE data were identified through breast MRI examinations captured within OMOP. BPE values were extracted directly from the corresponding radiology reports, where BPE is routinely documented as part of standardized clinical interpretation. We also recorded BPE from the most recent pre-treatment MRI report. BPE was qualitatively categorized as (1) minimal, (2) mild, (3) moderate, or (4) marked according to the breast imaging-reporting assessment and data system (BI-RADS). All MRI reports and their subsequent BPE classifications were independently determined by a board-certified breast radiologist.

BPE was dichotomized into low (1 and 2) versus high (3 and 4). Modeling all four BPE categories would substantially reduce power due to limited sample sizes within individual categories. Accordingly, BPE was dichotomized a priori into minimal or mild versus moderate or marked to ensure stable estimation and adequate statistical power. This dichotomization is consistent with prior studies and supports clinical interpretability. For example, a recent systematic review and meta-analysis compared diagnostic performance using the same categorization of BPE (minimal or mild vs. moderate or marked) [22].

Furthermore, neutrophil and lymphocyte counts were obtained from laboratory values of peripheral blood samples collected within eight weeks of breast cancer diagnosis, from 4 weeks before to 4 weeks after, preferentially using values closest to the time of diagnosis. This pre-treatment NLR was calculated by dividing the absolute neutrophil count by the absolute lymphocyte count.

All statistical analyses were performed using SAS version 9.4 (SAS Institute, Cary, NC, USA). Continuous variables were compared between IDC and ILC using *t*-tests, and categorical variables were compared using chi-square tests. Follow-up time was summarized by IDC and ILC subtype. Mortality outcomes (overall, 5-year, and 3-year) were also summarized by subtype.

Receiver operating characteristic (ROC) analyses were conducted to evaluate the association between the NLR and mortality separately within the IDC and ILC groups. Areas under the curve (AUCs) were estimated with 95% confidence intervals (CIs). Optimal NLR cutoffs were determined by maximizing the sum of sensitivity and specificity, and corresponding sensitivity and specificity values for mortality were reported.

NLR was summarized by BPE level (1–2 vs. 3–4) within IDC and ILC subtypes and compared using analysis of variance (ANOVA).

## 3. Results

Data from 794 patients was collected retrospectively, the characteristics of which are compiled in Table 1. A total of 693 (87%) patients had IDC while 101 (13%) patients had ILC.

### 3.1. Follow-Up Time and Mortality

Table 2 describes follow-up time in the study (years), summarized separately by group (IDC and ILC) and death status (Yes/No). Descriptive statistics (e.g., median, minimum, maximum) are presented to show how long participants were observed until either death (event) or censoring (survival at last contact or end of study).

Survivors have longer follow-up in average in both groups. However, the death group has a very small sample size; the data cannot reliably answer whether participants who survived tend to have different follow-up times than those who died.

Furthermore, we examined mortality at 3 and 5 years. Table 3 summarizes the number of deaths for each time interval. However, given the small sample size in the ILC group and the minimal difference between 3-year and 5-year mortality counts, separate survival analyses at these time points are not considered statistically meaningful or reliable.

### 3.2. Receiver Operating Characteristic (ROC) Analysis

**Figure 1:**
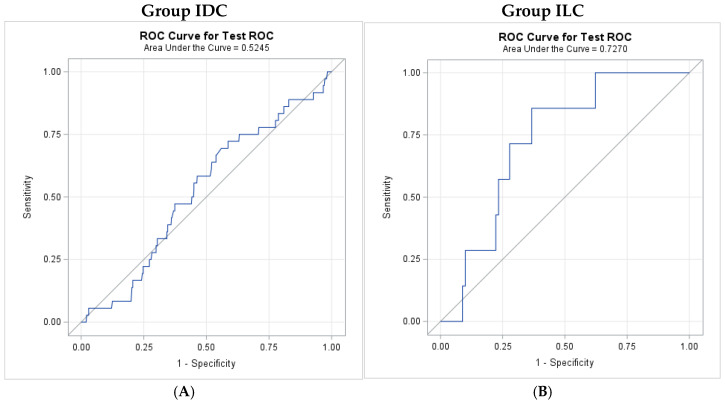
ROC analysis. (**A**). ROC curve for neutrophil/lymphocyte ratio (NLR) predicting mortality from invasive ductal carcinoma of the breast. The area under the curve (AUC) was 0.5245, indicating minimal predictive ability. (**B**). ROC curve for neutrophil/lymphocyte ratio (NLR) predicting mortality from invasive lobular carcinoma of the breast. The area under the curve (AUC) was 0.7270, indicating moderate predictive performance.

The NLR showed poor association with mortality in IDC (AUC = 0.525), and no optimal cutoff point could be estimated (Figure 1). In ILC, NLR demonstrated a significantly higher AUC of 0.727 (95% CI: 0.576–0.878) when compared with IDC (Figure 1). The associated optimal cutoff value of NLR in the ILC cohort is 2.13, yielding a sensitivity of 85.7% and specificity of 63.3% for mortality.

### 3.3. NLR and BPE

As seen in Table 4, ILC patients with high BPE showed the highest NLR overall. Specifically, the ILC group with high BPE had a mean NLR of 3.04, whereas the other three groups demonstrated significantly lower values: IDC with low BPE (mean = 2.10), ILC with low BPE (mean = 1.94), and IDC with high BPE (mean = 2.26). All pairwise comparisons were statistically significant (*p* < 0.05).

## 4. Discussion

The NLR has emerged as a promising biomarker in breast cancer, reflecting systemic inflammation that may influence tumor behavior and prognosis. While previous studies have examined the role of NLR in breast cancer, the biological differences between IDC and ILC suggest that these two subtypes warranted separate analyses. No prior study has examined the relationship of NLR to prognosis in the two subtypes separately, which may explain why elevated NLR associated with worse prognosis seen in some studies has not been consistently reproduced.

In our study, we stratified by ILC and IDC and examined the relationship of their NLR values to BPE and mortality separately. We found that the mean NLR was significantly higher in patients with ILC with high BPE compared to all other groups (IDC with high BPE, IDC with low BPE, and ILC with low BPE). When evaluating mortality using an ROC curve, although with a small sample size, it was found that NLR demonstrated a significantly higher AUC than IDC, with an associated optimal cut-off NLR of 2.13 with a sensitivity of 85.7% and specificity of 63.3% for mortality.

In this discussion, we will address the distinct nature of IDC and ILC, examine the clinical implications of our findings, and propose directions for future research.

### 4.1. NLR and Breast Cancer Subtype: IDC and ILC

The NLR, a simple ratio of absolute neutrophil/lymphocyte count in peripheral blood, has been linked to inflammation-driven mechanisms that promote tumor progression in various cancers, including breast cancer. Elevated NLR levels have been associated with poor prognosis, increased tumor aggressiveness, and a higher metastatic potential in breast cancer [4,5,6,7,8,9,10,11,12,13,14]. These associations suggest that NLR could serve as a non-invasive, cost-effective biomarker for monitoring tumor progression in breast cancer.

Inflammation plays a crucial role in the tumor microenvironment, with neutrophils contributing to both local tissue damage and immune evasion. Elevated neutrophil levels can promote tumor growth by enhancing angiogenesis, suppressing apoptosis, and facilitating metastasis. Conversely, lymphocytes, particularly T cells, are important for anti-tumor immunity, and their reduction may impair the body’s ability to control tumor progression. Thus, the NLR serves as a reflection of the systemic inflammatory response, with a higher ratio indicative of a tumor-promoting, inflammatory microenvironment, which could lead to a worse prognosis.

Several studies have linked increased NLR to worse clinical outcomes in breast cancer. For example, Chae et al. demonstrated that pre-NAC NLR predicts pCR and recurrence in triple-negative breast cancer (TNBC) [4]. A meta-analysis of 19 studies that evaluated the impact of pre-treatment NLR on outcomes in 5504 breast cancer patients found an association of elevated NLR with poor pCR, disease-free survival, and overall survival irrespective of clinical stage, nuclear grade, and Ki67 expression [14]. In a subgroup analysis, NLR was only associated with pCR in TNBC patients and not HR+/HER2- patients [14]. Despite research supporting NLR as a biomarker for prognosis, one investigation of nearly 1000 women with early breast cancer suggested lack of correlation between NLR and biological factors relevant for tumor progression [16].

Given the mixed findings in the literature and lack of investigations that separately analyze ILC and IDC in association with NLR, our research offers a novel perspective on the topic. It is now widely accepted that ILC and IDC are biologically distinct tumors, differing in several keyways, including their growth patterns, molecular characteristics, metastatic patterns, and responses to treatment. IDC and ILC represent the two most common histological subtypes of breast cancer. IDC, the most prevalent subtype, arises from the ductal epithelium while ILC originates from the lobular epithelium. ILC differs from IDC in that ILC is often less detectable on imaging, tends to grow more slowly, and is associated with a distinct pattern of metastasis, often involving the peritoneum, ovaries, and gastrointestinal tract [23]. Thus, these biological differences suggest that the systemic inflammatory response, as reflected by NLR, may also vary between these subtypes.

Our findings support our hypothesis and indicate that patients with higher NLR tend to exhibit more prominent BPE on breast MRI in patients with ILC, when comparing to all other groups, including ILC with low BPE, IDC with high BPE, and IDC with low BPE, as seen in Table 4. This association was not observed in IDC patients. Likewise, in Figure 1, NLR showed a poor association with mortality in IDC while NLR demonstrated a higher AUC in ILC. Although the discriminatory performance was moderate in the ILC cohort, an AUC of 0.727 suggests that NLR may provide utility for risk stratification in ILC when combined with established clinical and imaging factors, rather than serving as a standalone diagnostic tool. Overall, our findings on the relationship of NLR to BPE and mortality, separately, support the stratification of tumor subtypes when attempting to predict prognosis in breast cancer.

This observation suggests that the systemic inflammatory response reflected by NLR could influence breast tissue characteristics, including the degree of enhancement. A potential hypothesis-generating explanation for this relationship may involve interactions between inflammation and vascularity. Neutrophils, as part of the inflammatory response, can increase vascular permeability and enhance angiogenesis, which may explain the higher BPE seen in individuals with elevated NLR. These factors could adversely affect the tumor microenvironment and create favorable conditions for breast cancer growth and spread, which may impact ILC preferentially when associated with elevated NLR.

Furthermore, the presence of BPE in breast MRI is thought to be a manifestation of increased microvascular density within the fibroglandular tissue and has been shown to be affected by hormonal influences, age, and breast density, which could reflect underlying inflammation and cellular activity within the breast tissue. BPE has been associated with breast cancer risk, recurrence, and treatment outcomes [24,25,26,27,28]. The correlation between NLR and BPE may, therefore, provide additional insight into the inflammatory processes affecting both the tumor microenvironment and the surrounding breast tissue. Studying ILC and IDC separately in further research may help clarify the precise role of NLR in predicting outcomes and guide subtype-specific management strategies.

### 4.2. Clinical Implications: A Stratified Approach

The potential relationship between NLR, BPE, and mortality could have significant clinical implications in the context of a specific breast cancer subtype. For instance, patients diagnosed with ILC with high NLR and significant BPE might warrant closer monitoring or more aggressive diagnostic workup, especially if other risk factors for breast cancer are present. In addition, NLR could aid in the stratification of patients for more personalized treatment plans, particularly in identifying those at higher risk for aggressive disease.

Given the non-invasive nature of NLR measurement and its availability from routine blood tests, it could be seamlessly incorporated into clinical practice to complement imaging findings. Further studies examining the interplay of NLR with other imaging biomarkers and clinical factors are essential to develop a more comprehensive risk stratification model.

The potential clinical impact of NLR as a prognostic and predictive biomarker in breast cancer are significant, but our study suggests that it must be considered in the context of tumor subtype. For instance, in patients with ILC, high NLR could potentially indicate a more aggressive tumor with worse prognosis and higher likelihood of metastasis, making it a valuable tool for risk stratification and treatment planning.

In clinical practice, our results suggest that incorporating NLR into the decision-making process for breast cancer patients would require considering tumor subtype. For example, based on our results, patients with ILC and high NLR levels could benefit from more intensive monitoring and potentially more aggressive therapeutic strategies whereas NLR may not be as useful as a tool in patients with IDC. NLR can be incorporated into multimodal machine learning algorithms to potentially predict prognosis and outcomes more accurately.

Similarly, the relationship between NLR and BPE on MRI could assist in identifying high-risk patients for ILC. More prominent BPE combined with a high NLR could be a sign of a more inflammatory and aggressive tumor microenvironment, warranting close follow-up and potentially more advanced imaging.

### 4.3. Limitations and Future Directions

Several limitations must be considered. First, our study is a retrospective study, which limits our ability to draw conclusions about causality or the temporal relationship between NLR and BPE. Second, adjustment for potential confounders is constrained by missing data and a reduced effective sample size. Adjustment for stage, pathological T/N/M, and ER/PR/HER2 would result in approximately 38%, 45%, and 20% reductions in analyzable sample size, respectively. Consequently, multivariable regression with covariate adjustment would entail substantial information loss and an increased risk of model instability. Accordingly, given the exploratory nature of this study, the analyses are intended to be primarily descriptive rather than confirmatory. Further longitudinal studies with larger sample sizes are essential to better understand the role of NLR as a biomarker in breast cancer.

Additionally, we recognize that our AUC of 0.727 in the ILC group reflects moderate discrimination, and the wide confidence interval likely reflects the small sample size of the ILC subgroup, which in turn also constrains statistical power and generalizability. It should be noted that the observed AUC is consistent with prior studies evaluating NLR in similar contexts [29,30], supporting the external validity of our findings. The proposed cutoff was derived using ROC-based methods to balance sensitivity and specificity and is intended to provide risk stratification instead of definitive clinical decision-making. Given the exploratory nature of this analysis and the moderate discrimination observed, the cutoff should be interpreted as a data-driven reference point that may inform future studies. We emphasize the need for validation and replication in larger cohorts to further investigate our findings.

Another limitation lies in the subjective nature of BPE assessment, which can vary based on radiologist experience and imaging protocol. It should be noted that because this was a retrospective study relying on clinical report data from original MRI reports, there may be inter-reader variability between radiologists in determining BPE. However, our approach reflects real-world clinical practice, which supports the external validity of our study findings. The development of standardized methods for assessing BPE, potentially aided by machine learning and automated image analysis, would help reduce variability and improve the accuracy of BPE measurements in future studies.

Finally, the role of other inflammatory markers, such as C-reactive protein and cytokines, in conjunction with NLR, could provide a more comprehensive picture of the systemic inflammatory response in breast cancer. Future studies should explore these markers and their relationship with both IDC and ILC to develop a more nuanced risk stratification model.

## 5. Conclusions

When stratifying by ILC and IDC and examining the relationship of their NLR values to BPE and mortality, separately, we found a significant difference between the two subtypes. ILC with high BPE had a higher mean NLR compared to all patient groups in our study. Furthermore, although our sample size was small, when evaluating association with mortality, NLR had a significantly higher AUC in ILC compared to IDC, yielding a sensitivity of 85.7% and a specificity of 63.3% for mortality.

In conclusion, our study supports the growing body of evidence suggesting that the NLR is a valuable and readily available biomarker in breast cancer, correlating with tumor aggressiveness and advanced disease stages. However, our findings are novel in that they support that this correlation to BPE and mortality, separately, is breast cancer histologic-subtype-dependent, as it was only seen with ILC, reflecting the complex and intricate role neutrophils play in mediating tumor progression selectively. Moreover, our findings suggest a potential link between NLR and BPE on breast MRI, which appears to be breast cancer subtype-specific, highlighting the role of systemic inflammation in shaping both tumor characteristics and the surrounding breast tissue microenvironment. Future research, particularly large-scale, prospective studies, is needed to validate our findings, refine their clinical applicability, and further elucidate the clinical relevance of NLR in breast cancer diagnosis and management.

## Figures and Tables

**Table 1:** Characteristics.

	IDC *n* = 693 (87%)	ILC *n* = 101 (13%)	*p*-Value
**Age**	60.1 (11.4)	67.9 (9.7)	<0.0001
**Age at diagnosis**	55.7 (11.3)	63.5 (9.6)	<0.0001
**Clinical grade**			<0.0001
1	42 (8.5%)	5 (7.5%)	
2	225 (45.4%)	62 (92.5%)	
3	229 (46.2%)	-	
**Clinical T stage**			0.470
1	333 (51.3%)	57 (57.0%)	
2	235 (36.2%)	29 (29.0%)	
3	56 (8.6%)	11 (11.0%)	
4	25 (3.9%)	3 (3.0%)	
**Clinical N**			0.138
0	470 (70.1%)	80 (80.0%)	
1	168 (25.1%)	16 (16.0%)	
2	14 (2.1%)	3 (3.0%)	
3	18 (2.7%)	1 (1.0%)	
**Clinical M**			0.479
0	663 (98.1%)	97 (97.0%)	
1	13 (1.9%)	3 (3.0%)	
**Pathological T**			<0.0001
0	19 (4.3%)	1 (1.2%)	
1	296 (67.1%)	41 (48.8%)	
2	114 (25.9%)	24 (28.6%)	
3	11 (2.5%)	18 (21.4%)	
4	1 (0.2%)	-	
**Pathological N**			0.031
0	326 (73.4%)	61 (72.6%)	
1	98 (22.1%)	13 (15.5%)	
2	16 (3.6%)	7 (8.3%)	
3	4 (0.9%)	3 (3.6%)	
**Pathological M**			0.324
0	452 (98.9%)	88 (100.0%)	
1	5 (1.1%)	-	
**Therapy timing**			0.0007
Systemic therapy before surgery	145 (20.9%)	7 (6.9%)	
Systemic therapy after surgery	430 (62.0%)	81 (80.2%)	
Systemic therapy before and after surgery	118 (17.0%)	13 (12.9%)	
**NAC**			0.0004
No	430 (62.0%)	81 (80.2%)	
Yes	263 (38.0%)	20 (19.8%)	
**Post Rx Pathological T**			0.009
0	66 (35.9%)	1 (16.7%)	
1	81 (44.0%)	1 (16.7%)	
2	24 (13.0%)	4 (66.7%)	
3	11 (6.0%)	-	
4	2 (1.1%)	-	
**Post Rx Pathological N**			0.081
0	140 (72.2%)	3 (50.0%)	
1	36 (18.6%)	1 (16.7%)	
2	12 (6.2%)	2 (33.3%)	
3	6 (3.1%)	-	
**Post Rx Pathological M**			0.804
0	195 (99.0%)	6 (100.0%)	
1	2 (1.0%)	-	
**ER**			<0.001
+	503 (73.1%)	98 (98.0%)	
−	185 (26.9%)	2 (2.0%)	
**PR**			0.004
+	420 (61.0%)	76 (76.0%)	
−	268 (39.0%)	24 (24.0%)	
**HER2**			0.0003
+	146 (21.7%)	6 (6.2%)	
−	527 (78.3%)	91 (93.8%)	
**Tumor subtypes**			
ER−/HER2+ (±PR+)	53 (7.6%)	1 (1.0%)	0.013
ER+/HER2+ (±PR+)	90 (13.0%)	5 (5.0%)	0.020
ER+/HER2− (±PR+)	395 (57.0%)	90 (89.1%)	<0.0001
ER−/HER2− (PR−) (triple neg)	127 (18.3%)	1 (1.0%)	<0.0001
**Survival**			0.557
0 Dead	38 (5.5%)	7 (6.9%)	
1 Alive	655 (94.5%)	94 (93.1%)	

**Table 2:** Follow-up time in the study (years).

Group	Death	*N*	Median	Minimum	Maximum
**IDC**	No	655	3.23	0.18	7.65
	Yes	38	2.38	0.52	5.09
**ILC**	No	94	3.22	0.45	7.24
	Yes	7	2.48	0.57	6.56

**Table 3:** Mortality.

	IDC *n* = 693 (87%)	ILC *n* = 101 (13%)
**Overall Mortality**	38 (5.5%)	7 (6.9%)
**5-Year Mortality**	37 (5.3%)	5 (5.0%)
**3-Year Mortality**	28 (4.0%)	4 (4.0%)

**Table 4:** Comparisons of NLR across group and BPE categories.

	Low BPE		High BPE	
	IDC (*n* = 396)	ILC (*n* = 62)	IDC (*n* = 236)	ILC (*n* = 37)
**Mean NLR (Standard Deviation)**	2.10 (1.30)	1.94 (1.84)	2.26 (1.52)	3.04 (4.74)
***p-*** **value**	0.0027	0.0033	0.0147	Reference

## Data Availability

The data presented in this study are available on request from the corresponding author due to privacy and ethical restrictions.

## Abbreviations

The following abbreviations are used in this manuscript:

NLR	Neutrophil/lymphocyte ratio
ILC	Invasive lobular carcinoma
IDC	Invasive ductal carcinoma
BPE	Background parenchymal enhancement
MRI	Magnetic resonance imaging
BI-RADS	Breast imaging-reporting assessment and data system
EMR	Electronic medical records
NC	North Carolina
OMOP	Observational medical outcomes partnership
ANOVA	Analysis of variance
NAC	Neoadjuvant chemotherapy
ER	Estrogen receptor
HER2	Human epidermal receptor 2
HR	Hormone receptor
AUC	Area under the curve
ROC	Receiver operating characteristic
TNBC	Triple negative breast cancer
